# Electrode Separators for the Next-Generation Alkaline Water Electrolyzers

**DOI:** 10.1021/acsenergylett.3c00185

**Published:** 2023-03-27

**Authors:** David Aili, Mikkel Rykær Kraglund, Sinu C. Rajappan, Dmytro Serhiichuk, Yifan Xia, Valadoula Deimede, Joannis Kallitsis, Chulsung Bae, Patric Jannasch, Dirk Henkensmeier, Jens Oluf Jensen

**Affiliations:** † Department of Energy Conversion and Storage, Technical University of Denmark, Elektrovej, Building 375, 2800 Lyngby, Denmark; ‡ Department of Chemistry, University of Patras, 26504, Patras, Greece; § Department of Chemistry and Chemical Biology, Rensselaer Polytechnic Institute, Troy, New York 12180, United States; ∥ Polymer & Materials Chemistry, Department of Chemistry, Lund University, 221 00 Lund, Sweden; ⊥ Hydrogen·Fuel Cell Research Center, Korea Institute of Science andTechnology, Seoul 02792, Republic of Korea; # Division of Energy & Environment Technology, KIST School, University of Science and Technology, Seoul 02792, Republic of Korea; g Green School, Korea University, Seoul 02841, Republic of Korea

## Abstract

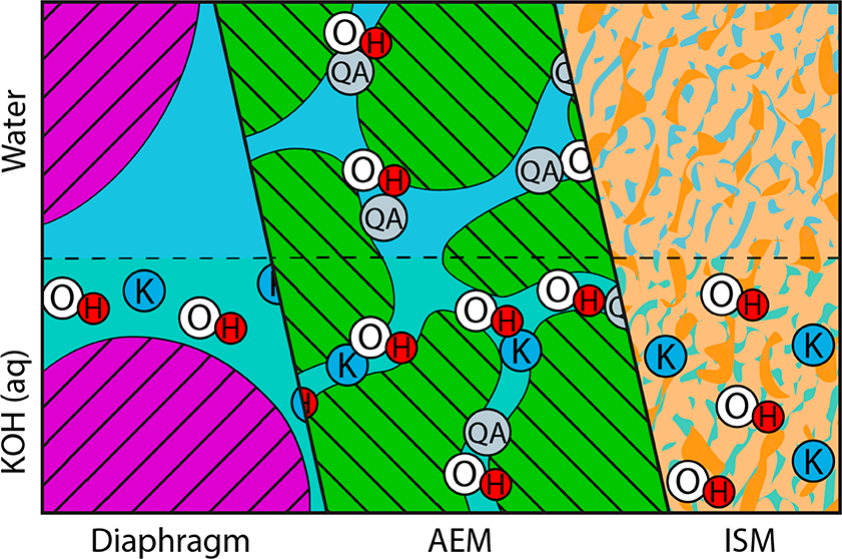

Multi-gigawatt-scale hydrogen production by water electrolysis is central in the green transition when it comes to storage of energy and forming the basis for sustainable fuels and materials. Alkaline water electrolysis plays a key role in this context, as the scale of implementation is not limited by the availability of scarce and expensive raw materials. Even though it is a mature technology, the new technological context of the renewable energy system demands more from the systems in terms of higher energy efficiency, enhanced rate capability, as well as dynamic, part-load, and differential pressure operation capability. New electrode separators that can support high currents at small ohmic losses, while effectively suppressing gas crossover, are essential to achieving this. This Focus Review compares the three main development paths that are currently being pursued in the field with the aim to identify the advantages and drawbacks of the different approaches in order to illuminate rational ways forward.

Hydrogen (H_2_) produced by water electrolysis is central in the green transition, both as a storage medium of energy and as a feedstock for the production of sustainable fuels, chemicals, and materials.^1^ Installations at the multi gigawatt scale will be needed to meet the global demands for green H_2_,^2^ and the alkaline water electrolysis technology plays a key role in this connection. This is primarily because the scale of implementation is not limited by the availability of critical raw materials, in contrast to the proton exchange membrane (PEM) electrolyzers, which depend on electrocatalysts and coatings containing platinum and iridium.^3^

Alkaline water electrolysis is a mature technology that has been available on a commercial basis for more than a century, but the new technological context of the renewable energy system introduces new requirements in terms of energy efficiency, rate capability, response time to load changes, and pressureability.^4,5^ The first developed cell design makes use of two-dimensional nickel-based electrodes, separated by a porous diaphragm that enables ionic contact between the electrodes while suppressing intermixing of the product gases. The evolved gases are discharged through the electrolyte-filled gap between the electrodes and the separator. With this design, an interelectrode distance in the range of several millimeters is inevitable. This results in area-specific resistance (ASR) exceeding 1–2 Ω cm^2^,^6^ which from an energy efficiency point of view hardly justifies H_2_ production rates higher than 200–400 mA cm^−2^ (corresponding to around 100–200 N mL_H_2__ cm^–2^ h^–1^). Bringing the electrodes closer to each other in a zero-gap configuration is the most obvious strategy to reduce the ASR, hence increase the rate capability, but this requires fundamental redesign of components and cell architectures since the produced gases need to be discharged to the backside of the electrodes. At the materials level, this development direction calls for geometries that can drive the electrochemical reactions at low overpotentials and allow for efficient mass transport and fast gas discharge. The development trends and progress with respect to catalysts, electrodes, and cell designs are discussed in recent excellent reviews.^7−9^ The ASR of the current state-of-the-art (SoA) cells is around 0.25 Ω cm^2^,^10^ which will be discussed further in the coming paragraphs.

To fully capitalize on the zero-gap cell concepts and the new highly active three-dimensional electrode chemistries and geometries, there is a need for new electrode separators with excellent stability that can support high currents at small ohmic losses, while effectively suppressing crossover of evolved gases, even at high pressure, and promoting gas discharge to the backside of the electrodes. In this Focus Review, we discuss the different electrolyte materials design strategies that are currently being pursued in the research field in the light of (1) ion conductivity and ASR, (2) gas crossover, and (3) chemical/mechanical stability. Efficiency and cost at the system level depend strongly on these parameters but are not analyzed in detail in this work.

Figure 1 illustrates schematically the three main electrolyte concepts that are currently being applied in relation to alkaline water electrolysis, and the typical chemical structures of SoA materials used in the different approaches are provided in Figure 2. The porous diaphragms (Figure 1a) are typically prepared from poly(arylene ether sulfone)-bonded inorganic hydrophilic particles by a phase inversion casting process to form a highly porous composite material with typical pore size at the micrometer scale and with denser surface layers.^11^ Poly(phenylene sulfide) is also used in the form of fibers, but requires alternative processing routes due to the insolubility in organic solvents.^12^ The anion-exchange membrane (AEM) approach (Figure 1b) is based on polymer chemistries equipped with covalently attached cationic functionalities, and membranes derived from quaternary ammonium-functionalized poly(arylene ether sulfone)s were among the first AEM chemistries used for electrolysis device tests.^13,14^ Specific examples of the most successful cationic ionomers or ionenes with demonstrated device performance at the single-cell level so far include structures derived from polystyrene, poly(arylene benzimidazolium), poly(arylene piperidinium), poly(arylene alkylene), and polyphenylene.^15−17^ At the stack level, systems based on polycarbazole membranes have also been reported.^18^ When soaked in water, the ionic groups dissociate into the immobile cationic groups on the polymer and mobile hydroxide ions that support the ionic current between the electrodes. Like perfluorosulfonic acid membranes, many AEM chemistries have been reported to develop a phase separated nanomorphology upon hydration, although structure–property relationships depend on a large number of parameters and are not yet fully understood.^19^ The third approach (Figure 1c) makes use of ion-solvating polymer membranes that contain polar or ionizable groups, such as benzimidazoles or imidazoles, that promote excessive uptake of aqueous alkali metal hydroxide solutions to form a homogeneous morphology without distinct phase separation.^20−22^ The term ion-solvating polymer electrolyte was introduced in the context of nonaqueous electrolyte chemistries based on mixtures of, e.g., poly(ethylene oxide) and lithium salts but has been adapted by the alkaline polymer electrolyte community for polymer systems doped with aqueous KOH although excess water needs to be present to fully solvate the dissociated ions.^23^

**Figure 1:**
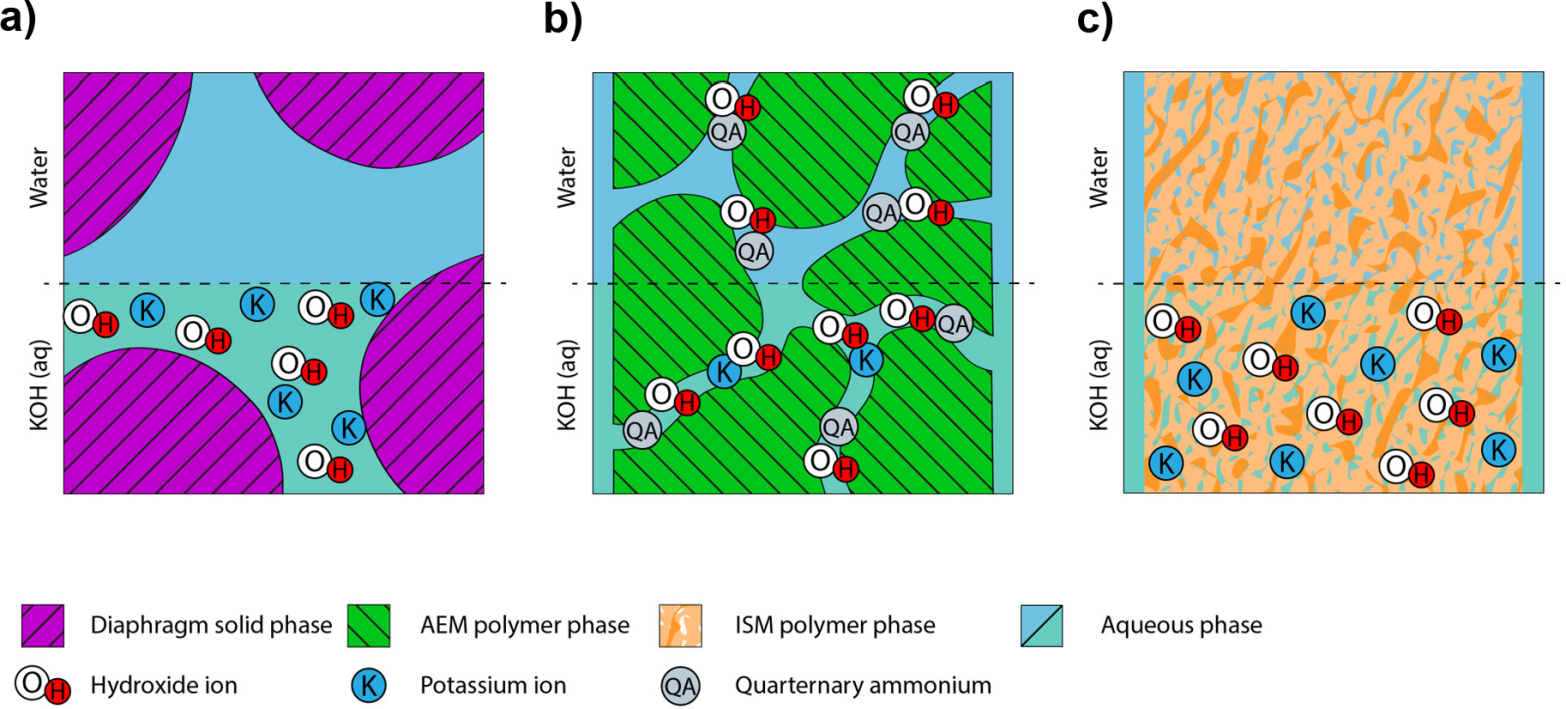
Schematic illustration of the different electrolyte concepts in pure water and combined with aqueous KOH based on (a) porous diaphragms, (b) AEM, and (c) ion-solvating membranes. The dimensions of the electrolyte filled pores in the bulk of SoA porous diaphragms (a) is typically in the micrometer range or slightly smaller. A general understanding of the nanomorphology of AEMs (b) has not been established, but a phase separated morphology at the nanometer length scale has been confirmed experimentally for some materials. The ion-solvating membranes (c) are generally understood as more homogeneous systems.

**Figure 2:**
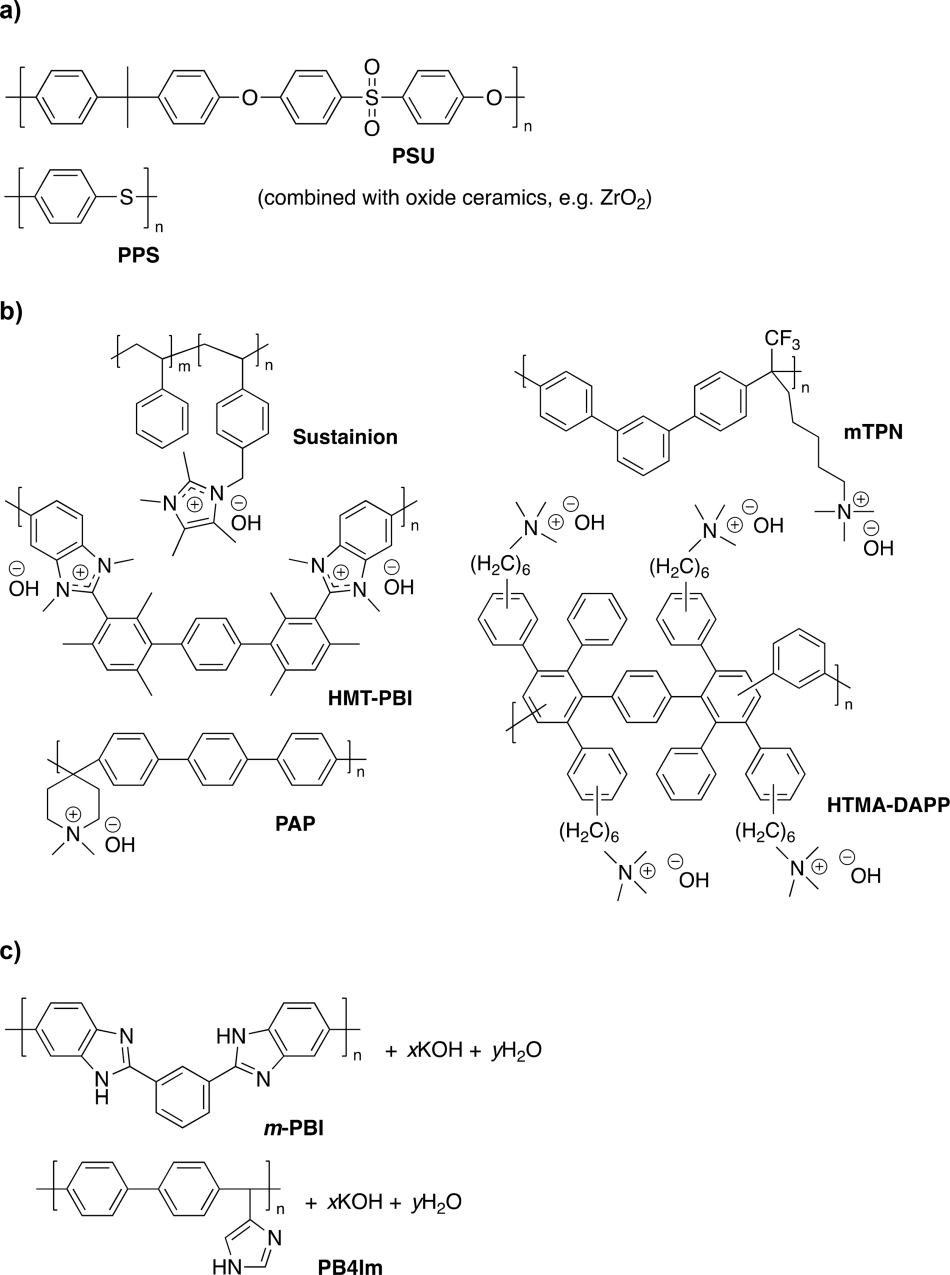
Examples of SoA polymer chemistries used for (a) porous diaphragms, (b) AEM in pure water and combined with an aqueous KOH support electrolyte, and (c) ion-solvating membranes.

Figure 3 compares polarization data for best-performing SoA cells constructed based on non-noble metal electrodes combined with a PSU/ZrO_2_ composite porous diaphragm,^10^ a quaternary ammonium functionalized polyphenylene-derived AEM operated in pure water,^15^ an imidazolium functionalized polystyrene-derived AEM combined with a 1 mol L^–1^ KOH support electrolyte,^24^ and finally a polybenzimidazole-based (*m*-PBI) ion-solvating membrane.^25^ The slopes of the linear parts of the polarization curves in the 1000–1600 mA cm^–2^ range correspond to an ASR of 0.25, 0.28, 0.14, and 0.17 Ω cm^2^ for the cells based on the porous diaphragm, AEM in pure water, AEM combined with support electrolyte and the ion-solvating membrane, respectively. An overview of the key characteristics for the cells based on the different separator concepts is provided in Table 1.

**Table 1:** Comparison of Conditions and Key Characteristics for Noble-Metal-Free SoA Cells Based on Porous Diaphragms,^10^ AEM Combined with Pure Water,^15^ AEM Combined with Aqueous Support Electrolyte,^24^ and Ion-Solvating Membranes^25^ Shown in Figure 3

	**Porous diaphragm**	**AEM combined with pure water**	**AEM combined with support electrolyte**	**Ion-solvating membrane**
Conditions	30% KOH, 80 °C	0% KOH (pure water), 85 °C	5% KOH (1 mol L^–1^)	24% KOH, 80 °C
Anode	NiFe LDH^a^ on Ni foam	NiFe on Ni nanofoam	NiFe on steel felt	NiAl on Ni mesh
Cathode	NiAl on Ni foam	NiMo on carbon	NiFeCo on carbon	NiAlMo on Ni mesh
Separator material (see Figure 2)	Porous PSU/ZrO_2_ composite	HTMA-DAPP	Sustainion	*m*-PBI
Separator thickness	300 μm	26 μm	50 μm	150–200 μm
ASR^b^	0.25 Ω cm^2^	0.28 Ω cm^2^	0.14 Ω cm^2^	0.17 Ω cm^2^
Ex situ conductivity	120 mS cm^–1^	120 mS cm^–1^	115 mS cm^–1^	>200 mS cm^–1^ (at RT)
In situ conductivity^c^	120 mS cm^–1^	9 mS cm^–1^	36 mS cm^–1^	88–118 mS cm^–1^
H_2_ in O_2_ level	0.3–1.2% at 50 mA cm^–2^	Not reported	Not reported	1.2% at 50 mA cm^–2^
	0.25% at 300 mA cm^–2^			0.1% at 1000 mA cm^–2^

a Layered double hydroxide.

b Calculated from the linear slopes of the polarization curves in Figure 3 in the 1000–1600 mA cm^–2^ range.

c Calculated from the ASR and reported thickness.

**Figure 3:**
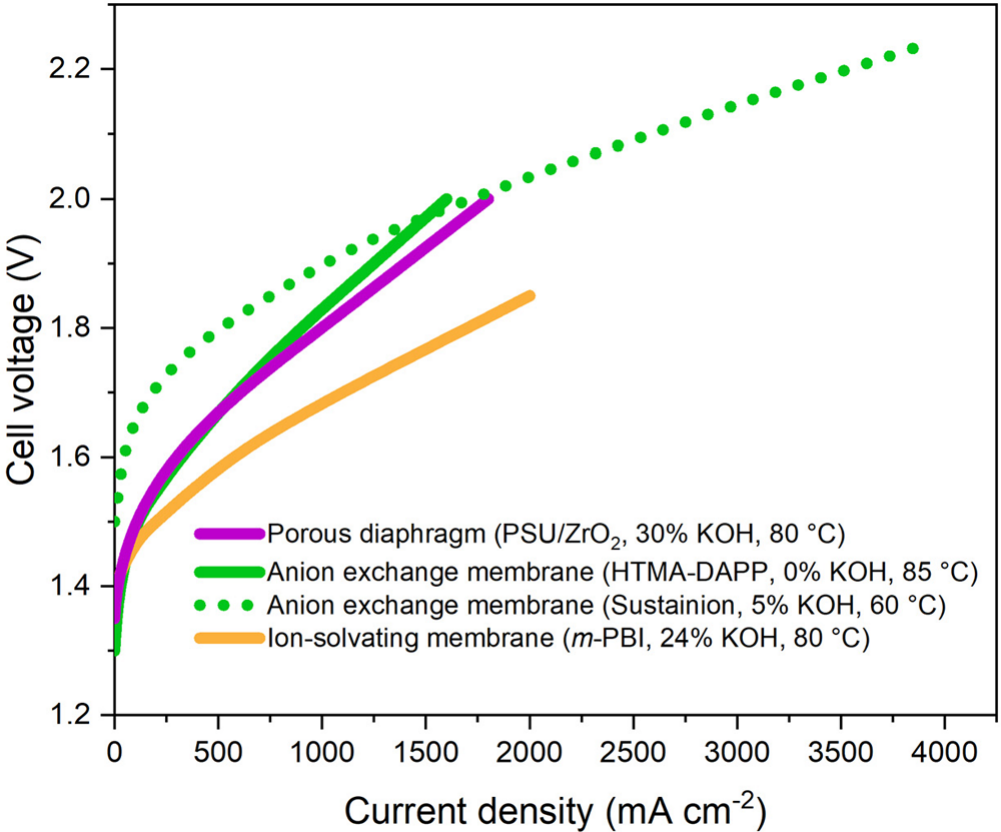
Comparison of polarization data for current SoA cells equipped with noble-metal free electrodes and a porous diaphragm,^10^ an AEM in pure water,^15^ an AEM combined with KOH support electrolyte,^24^ and an ion-solvating membrane.^25^ The polarization curves have been reconstructed based on data points extracted from the original references. The reader is referred to the original references for further details about cell components, hardware, and operating parameters.

For the porous diaphragm, the ASR obtained from the linear fit of the polarization curve in the high current density range was identical to the ASR recorded by AC impedance in a H-cell. For the AEM based cell, on the other hand, the conductivity of the membrane alone was about an order of magnitude higher than the conductivity calculated from the ASR at the cell level. However, it should be noted that the ASR decreased from 0.28 Ω cm^2^ to 0.06 Ω cm^2^ (corresponding to 43 mS cm^–1^) when the cell was operated with a liquid KOH feed instead of pure water. The performance benefits of operating with an aqueous KOH support electrolyte are also well-documented by the comprehensive study by Zignani et al.^26^ for AEM cells equipped with various nickel-based electrode chemistries. It should also be noted that current densities far exceeding 4000 mA cm^–2^ at <1.9 V have been reported for AEM cells, but this has so far only been achieved when combined with precious metal electrodes and alkaline support electrolytes to govern both catalyst kinetics and ion conductivity.^27^

## Conductivity and ASR

The primary function of the electrode separator is to enable ionic contact with high hydroxide conductivity between the electrodes, so that high currents can be conducted at small ohmic voltage losses. The heterogeneous diaphragms are based on dimensionally stable porous matrix materials, where the ion conductivity is provided by the aqueous electrolyte that fills the volume of the percolating pores. The ionic resistance across the diaphragm therefore depends on the conductivity of the aqueous electrolyte solution and a number of geometric parameters (thickness, porosity, and tortuosity), as well as the wettability of the porous matrix interior.^28^ For the commercially available Zirfon Perl UTP 500 diaphragm, the ASR in 30 wt% aqueous KOH (aq.) is reported to be as low as 0.3 and 0.1 Ω cm^2^ at 20 and 80 °C, respectively.^29^ This diaphragm has a thickness of 500 μm, which means that the ASR translates to a specific conductivity of 167 and 500 mS cm^–1^ in 30 wt% KOH at 20 and 80 °C, respectively. For comparison, the specific conductivity of 30 wt% KOH at 80 °C is close to 1400 mS cm^–1^.^30^ However, the ASRs reported for Zirfon vary largely in the literature and seem to depend on the measurement methodology, sample history, electrode selection, and eventually also on bubble nucleation within the pores during operation leading to partial blanketing of the ionic pathways.^29^

The conductivity of AEMs is mediated by covalently attached ionic groups, typically quaternary ammonium hydroxides, which dissociate upon hydration leading to sorption of excess water. The hydroxide conductivity depends on the specific membrane chemistry, ion-exchange capacity (IEC), and degree of hydration, and can exceed 100 mS cm^–1^ in pure water for optimized materials derived from, e.g., poly(arylene alkylene)s,^31^ poly(arylene piperidinium)s,^32,33^ poly(arylene benzimidazolium)s,^34,35^ poly(arylene imidazolium)s,^36^ radiation-grafted polyolefins,^37^ or polyphenylenes.^38^ Even though the conductivity of AEMs can be remarkably high in pure water, the ASR at the cell level with a pure water feed is often considerably higher than the ASR of the membrane alone.^39^ For a well-functioning cell, the ohmic resistance across the electrode separator is generally the main contributor to the total ASR. By comparing the ex situ conductivity of the membrane with the conductivity calculated from the ASR at the cell level, one can get a qualitative indication of how well the other cell components are working. For example, in Table 1 the ex situ conductivity of the porous separator matches the in situ conductivity calculated from the ASR during electrolysis tests. This indicates that the ohmic resistance in the electrodes and that at the separator–electrode interface are very small in relation to the total ohmic resistance. On the other hand, for the cell equipped with an AEM operated in pure water, the in situ conductivity calculated from the ASR is more than an order of magnitude lower than the ex situ conductivity. This indicates that ohmic resistance contributions from other cell components are dominating.

A common way to reduce additional voltage losses is to operate the cell with a dilute aqueous support electrolyte of 0.5–5 wt% KOH,^15,26^ suggesting that the predominating ohmic voltage losses originate from ionic resistance contributions within the electrodes or at the membrane–electrode interface. The conductivity of 5 wt% aqueous KOH at 80 °C exceeds 300 mS cm^–1^,^30^ and with such a support electrolyte present within the electrode and at the membrane–electrode interface, the conduction does not solely rely on having a well-dispersed and interconnected ionomer phase that provides ionic contact between the electrodes and the membrane. In this way the electrode design becomes less sensitive to defects and imperfections, and it may even extend the electrochemically active surface area to regions that are not contacted by the ionomer phase. It may also mitigate the impact of ionomer adsorption and poisoning of the electrocatalysts, which recently has been identified as a major challenge in connection to electrode development for AEM electrolysis.^40^ With proper electrode optimization, the sum of overpotentials can be reduced to a level where current densities of up to 1000 mA cm^–2^ can be supported in pure water with noble-metal-free electrodes at a cell voltage below 1.9 V (see Figure 3).^15^

The conductivity of ion-solvating membranes is provided by the electrolyte solution absorbed by the polymer matrix. The use of a conducting electrolyte is therefore required, which also implies that the contacting between the electrodes and the membrane is less challenging than for an AEM system operating in pure water. From a conductivity point of view, the ternary electrolyte system based on KOH (aq.)-doped *m*-PBI has proven particularly promising in this class of electrolytes, showing ion conductivity exceeding 100 mS cm^–1^ in 25 wt% aqueous KOH at 20–80 °C.^41^ Technologically relevant conductivities have also been achieved with modified polybenzimidazole chemistries^42−44^ or *m*-PBI blends with different anion exchange materials,^45,46^ polyisatine,^47^ or poly(vinyl alcohol).^48^ Polybenzimidazole-free systems based on polyvinylpyrrolidone–poly(arylene ether sulfone) blends,^49^ imidazole-functionalized poly(arylene alkylene)s,^22^ or perfluorosulfonic acids^50^ have also been evaluated. It should be remarked that the latter example is based on a sulfonated polymer, which shows remarkably high cation selectivity in pure water and at low degrees of hydration.^51^ However, upon excessive swelling in high ionic strength solutions the selectivity vanishes, and with increasing swelling and electrolyte uptake, the partial conductivities (transference numbers) of the different ionic species gradually approach those of homogeneous aqueous solutions as the dilution of the ionic sulfonate groups diminishes the Donnan exclusion.^52^ Similarly, the conductivity of AEMs containing aqueous support electrolytes is likely supported by a significant cationic contribution. Another effect is that the water content of membranes decreases when the ionic strength of the external solution increases. For example, several AEMs showed a higher in-plane conductivity in pure water than through-plane conductivity in 0.5 mol L^–1^ KOH solution, and again an increasing conductivity when the solution concentration was further increased, presumably because the decreased Donnan exclusion allowed for further uptake of KOH.^17^ One hypothesis that develops from this discussion is that the conductivity behavior of ion-exchange membranes functionalized with cationic or anionic groups and neutral hydrophilic ion-solvating membranes is mainly dictated by the excess absorbed electrolyte if the volume fraction of electrolyte within the membrane is high. However, this remains to be confirmed experimentally by quantification of the transference numbers of the different ionic species present and taking morphological effects and electrolyte volume fractions into consideration.

## Gas Crossover

Regardless which type of electrode separator is used, its second main function is to prevent the evolved gases from intermixing. Any crossover of H_2_ to the anode compartment lowers the Faradaic efficiency of the cell, and for safety reasons the H_2_ content in the anodic O_2_ stream must always be kept far below the ignition limit (about 4% H_2_ in air and presumably even less in O_2_).^53^ For conventional cells based on porous diaphragms, this becomes most critical during partial load operation and, in particular, when the O_2_ evolution rate decreases. Depending on the particular system engineering and electrolyte flow schemes, the H_2_ in O_2_ level in ambient pressure electrolyzers typically ranges from 1.5 to 2.5% at 50 mA cm^–2^ and decreases to 0.5–1.5% at 400 mA cm^–2^ due to dilution.^10^ As discussed by de Groot and Vreeman,^29^ the crossover of gas bubbles at low differential pressures is essentially negligible, since the diameter of the smallest H_2_ bubbles (around 20 μm) is at least an order of magnitude larger than the dimension of the largest pores. The mechanism that dominates the permeation is the crossover of electrolyte solution that is (super)saturated with dissolved H_2_ or O_2_. The driving force for this may be a combination of diffusion driven by concentration gradients or convection and the electroosmotic drag in the direction of the hydroxide ion current from the cathode toward the anode. The latter is an intrinsic transport mechanism connected to the vehicular contribution to the ion conduction, which in the alkaline environment results in a flux of aqueous electrolyte toward the O_2_ evolution electrode. As pointed out by Trinke et al.,^54^ this could potentially make H_2_ crossover an even larger challenge for AEM electrolyzers as compared with PEM systems, where the direction of the electroosmotic drag is from the O_2_ evolution electrode (anode) toward the H_2_ side (cathode). From a system engineering point of view, it is desirable to operate at pressures exceeding 30 bar to simplify the postcompression, which could lead to problematic H_2_ in O_2_ levels under partial load conditions due to the increased H_2_ solubility with increasing pressure.^55^

Molecular permeability is the product of solubility and diffusivity, and for H_2_ (and oxygen), both factors decrease with increasing KOH concentration. The H_2_ solubility at 30 °C is reported as 16.68, 14.13, 12.13, and 1.59 N cm^3^ H_2_ L^–1^ for aqueous KOH with concentrations of 0.0091, 0.51, 1.03, and 7.61 mol L^–1^, respectively.^56^ The nature of the ionic environment in the separator may well deviate from that of bulk KOH (aq.), but it is striking that H_2_ solubility is lower by the factors 12 and 7.7 in 7.61 mol L^–1^ KOH as compared to water and 1 mol L^–1^ solutions, respectively. From a crossover perspective, this implies that cell operation with support electrolytes of higher alkali concentration is an advantage.^57^ This is in conflict with the general materials design strategies that are typically outlined in the AEM electrolysis community, where focus is on reduced membrane thicknesses to minimize the ASR and on lowered concentration of the support electrolyte to reduce its corrosiveness.^16,58^ The crossover aspect is often overseen in this discussion. It should also be noted that the hydroxide ion concentration within the aqueous domains of hydrated AEMs is significant even when equilibrated in pure water, which also influences the solubility of gases. For example, the commercially available FAA3-50 AEM from Fumatech has a volume fraction of water of around 0.8 when fully hydrated,^59^ which corresponds to a hydroxide ion concentration exceeding 0.7 mol L^–1^ in the aqueous phase assuming an IEC of 2.1 mequiv g^–1^ and a dry polymer density of 1.44 g cm^–3^.

Crossover data are rarely reported along with cell test data in the scientific literature. Hence, more research is needed to fully understand how crossover depends on the electrolyte systems used, the design of the electrodes, and bubble nucleation and growth in the complex structures that are used. Crossover of H_2_ by diffusion in the opposite direction of the electroosmotic drag, enhanced by supersaturation of H_2_, is a well-known challenge in PEM electrolysis and is typically mitigated by using relatively thick membranes such as Nafion 117 (175 μm in the dry state) or by introducing recombination catalysts near the anode.^60^ Similar design aspects may be considered for AEM electrolyzers, in particular if very thin membranes are used and if the cell is pressurized and operated with a pure water feed or with a low concentration of support electrolyte. As the H_2_ solubility decreases with increasing ionic strength, one strategy to suppress undesired crossover could be to increase the IEC while maintaining relatively low degrees of hydration.

To counteract softening of the membranes and increase dimensional stability, many membranes developed for electrolysis include porous supports. A common problem is formation of voids along the porous support,^61^ which then increases the H_2_ crossover. Presumably, this effect should also be observed for water vapor permeation, which can be easily measured. For example, a reinforced FAA3-PK-75 AEM showed a higher water permeability than a non-reinforced FAA3-50 membrane, but a similar water flux, because the reinforced membrane was 50% thicker.^61^ One strategy to tackle this issue is to increase the interaction between the porous support and the ion-conducting matrix. For example, PBI nanofiber mats can be prefilled with bromoalkylated polymers, which then form covalent bonds with the PBI imidazole groups and are transferred into the quaternary ammonium form AEM in a final step.^17^ Similar developments are expected to be seen for other alkaline systems in the future, improving simultaneously dimensional stability and mechanical strength while suppressing H_2_ crossover.

## Chemical and Mechanical Stability

Highly alkaline environments are challenging for most polymeric materials due to various degradation pathways triggered by nucleophilic attack by the hydroxide ions. The PSU binder in typical porous diaphragms shows excellent chemical resistance as a bulk material in concentrated aqueous KOH at 80–90 °C for several years,^62^ but recent findings in the AEM community show that arylene ether linkages are intrinsically unstable in alkaline environment, especially if activated by nearby electron-withdrawing groups.^63^ The main reason for the good stability in the bulk form may thus be a result of the hydrophobicity of the polymer, which effectively keeps the hydroxide ions away from the vulnerable arylene ether moieties in the bulk material. Poly(arylene ether sulfone)s and other poly(arylene ether) derivatives^64,65^ were among the first backbone chemistries explored for application in AEM electrochemical devices, but the installation of tethered electron-withdrawing cationic groups along the main chain has been found to significantly enhance the rate of chain scission.^66^ The current trend is therefore to introduce longer alkyl spacers between the backbone and the cationic group,^67^ and to make use of backbone chemistries devoid of labile arylene ether linkages or other strongly polarized bonds. More robust choices are polyolefins,^37^ poly(arylene alkylene)s,^68^ polyfluorenes,^69^ or polyphenylenes.^38,70^ An alternative strategy to mitigate backbone degradation is to protect electrophilic positions in the backbone by steric hindrance by installing bulky functionalities in the vicinity of the labile groups. This has proven particularly effective for ionenes derived from poly(dialkylbenzimidazolium)s^34,35^ and poly(dialkylimidazolium)s.^36^ Other approaches make use of inductive effects to stabilize the backbone, for example by introducing electron donating functionalities near the electrophilic positions.^71^ The alkaline environment is also a formidable challenge when it comes to the stability of the cationic functionalities, which are prone to degrade due to various substitution and elimination reactions.^72−74^ While quaternary ammonium cations installed in the benzylic position rapidly degrade by substitution, cations with protons in the β-position suffer from Hofmann elimination. The former degradation mode can be suppressed by avoiding benzylic cations, and the latter can be circumvented by replacing the β-hydrogens with alkyl functionalities. Other common degradation mitigation strategies include steric protection^75^ (as also mentioned for cationic ionenes above), charge delocalization,^76^ and optimization of conformational or geometric features that contribute to increasing the activation barrier of common degradation pathways.^32,77^

Chemical stability at the membrane level is typically assessed by keeping the membrane in an aqueous alkaline solution at elevated temperature for extended durations, while monitoring eventual changes of weight, IEC, molecular weight and distribution, mechanical strength, conductivity, or molecular structure. The AEM chemistries based on poly(dialkylimidazolium)s show only minor signs of degradation from NMR data after 1 week in 40 wt% KOH at 100 °C,^36^ and no apparent signs of degradation could be detected by NMR after 4–5 days of steady-state operation at 200 mA cm^–2^ at 60 °C in 1 mol L^–1^ KOH.^78^ For AEM cells operating with pure water or with dilute KOH support electrolytes (e.g., 0.1 mol L^–1^), recent studies indicate that the stability of the ionomer binder in the catalyst layers is the most critical parameter to address to ensure stable ionic interfacial contact between the electrodes and the membrane.^58,79^

It is also important to stress that there can be a large discrepancy between the stability limits under simulated operating conditions and what is observed during operation in the electrolyzer. For example, ion-solvating membranes of polybenzimidazole show a lifetime in the range of several months in 25 wt% KOH at close to 90 °C but only 1–2 weeks in the electrolyzer operating in a similar KOH concentration and temperature range.^21,80^ By reinforcing the membrane, the lifetime can be extended to >1000 h, despite the intrinsic instability of the polymer matrix.^25^ One of the reasons is likely that the reinforcement contributes to the relief of mechanical stress caused by electrolyte flow, gas evolution, and uneven swelling, which for mechanically weak and thin membranes can be particularly problematic and result in premature membrane failure around the electrode edges.^21^ Recent model system studies indicate that the degradation of polybenzimidazoles primarily proceeds via the fraction of neutral benzimidazole units, which suggests that it may be possible to improve the backbone stability by increasing the acidity of the benzimidazoles by structural modifications.^81^ In combination with mechanical reinforcement and eventually steric hindrance, this could be an effective route toward durable and high-performing ion-solvating membranes based on polybenzimidazoles.

The mechanical properties of ion-solvating membranes based on *m*-PBI equilibrated in 6 mol L^–1^ KOH in terms of tensile strength (around 16 MPa) and tensile modulus (around 400 MPa) are similar to those of AEM membranes based on the polyphenylene (HTMA-DAPP) shown in Figure 2.^40,82^ However, the mechanical characteristics depend largely on the electrolyte or water uptake. For reinforced ion-solvating membranes, the mechanical characteristics of the composite membrane are dominated by the mechanical characteristics of the reinforcement material.^25^ For porous separators based on poly(arylene ether) bonded oxide ceramics, the introduction of a PPS reinforcement mesh has been demonstrated to increase the tensile strength of the composite materials from around 2 to 14 MPa.^83^

The availability of alkaline electrolysis test data that include information about membrane and separator stability is rather limited in literature, and the correlations between the stability observed under simulated test conditions and the stability determined from real cell tests remain to be investigated. The conditions that the membrane/separator experience inside the operating cell extend the number of stress factors to include potential difference, the presence of metal ions, radical formation, O_2_ exposure, mechanical stress due to pressure differentials, as well as local hot-spots due to uneven current distribution or H_2_/O_2_ recombination.

## Toward Electrode Separators for Next-Generation Alkaline Water Electrolysis

Table 2 summarizes key characteristics of the three types of separators. In conclusion, the main advantage of the porous diaphragm is that it is well-established and long-lasting. Moreover, it is independent of an ionomer phase in the catalyst layer. A down-side is its large thickness and the resistance it creates. For the AEM, main advantages are the low thickness that allows for compact stacks with high rate capability and the perspective for pure water operation, which reduces the corrosion-protective demands for the balance of plant materials. The H_2_ crossover aspect for AEM water electrolyzers is rarely discussed in the scientific literature but mitigating H_2_ crossover likely remains a challenge for AEM systems with thin membranes combined with low concentration of KOH supporting electrolytes. Differential pressure operation was recently explored by Motz et al.^40^ for AEM cells with up to 7 bar H_2_ at the cathode side, and the results seem to support that the H_2_ crossover needs to be further suppressed to allow for safe operation. The work also points out the importance of high mechanical strength and modulus for the longevity at the cell level, where a rigid backbone based on polyphenylene (HTMA-DAPP) was found to have a competitive edge as compared with more flexible materials based on poly(arylene alkylene)s. Another disadvantage of the absence of lye is that an ionomer needs to be incorporated into the catalyst layers in the electrodes. The experience, especially for alkaline fuel cells, is that this ionomer is one of the first components to suffer from degradation, and it makes electrode design and manufacturing more complicated. The ion-solvating membrane is in some sense a hybrid between the two former. Like the diaphragm, it works only with a concentrated alkaline electrolyte, so it shares the advantages and drawbacks related to this. With the AEM, it shares the potential for very low thickness and high rate capability, while the presence of a concentrated KOH electrolyte reduces H_2_ crossover and allows for ionomer-free electrodes. Since the principal challenge for new membranes is long-term stability, it is an advantage for the ion-solvating membranes that there is a broader selection of polymeric materials to search among or develop, since durable materials can be sought among cationic, anionic, and neutral polymers, as long as it possesses the right hydrophilicity to swell to the desired degree. Potential candidates include stabile backbone chemistries that have be identified in the field of AEMs, such as polyolefins, poly(arylene alkylenes), or polyphenylenes. Finally, integration of ion-solvating membranes in the SoA liquid alkaline electrolyzers operated with concentrated KOH requires only minor modifications of stacks and systems, and this may prove a big advantage leveraging implementation of the new technology especially on the short-term. A potential drawback of ion-solvating membranes based on anionic polymers could be the lower partial hydroxide conductivity in aqueous KOH as compared with cationic polymers at similar electrolyte volume fractions.

The development trend for porous diaphragms is to reduce the dimensions of the pores and the thickness to lower the ASR without compromising on H_2_ crossover resistance.^10^ Neutron scattering experiments on polybenzimidazole-based ion-solvating membranes indicate that the polymer directly after doping shows a chain separation range of about 10 to 20 nm.^20^ Reducing the pore size of the porous diaphragms to this level would thus imply that the two concepts meet. The AEM cells also clearly benefit on operating with a support electrolyte of 0.5–2 mol L^–1^ KOH, to improve electrode kinetics and contacting between the membrane and the electrodes. As discussed above, the conductivity of aqueous support electrolytes in that concentration range exceeds that of the ionomer phase, which makes the ionomer phase redundant. This seems supported by the recent studies by Wang et al.,^84^ who constructed AEM based cells with ionomer-free electrodes that showed remarkable performance when combined with a 1 mol L^–1^ support electrolyte. This development also brings the AEM concept closer to the ion-solvating membrane approach.

In a recent paper by Motealleh et al.,^85^ a hybrid membrane that combines a cationic polymer from the AEM community with ZrO_2_ nanoparticles used for porous diaphragms into a composite material that after doping with a 1 mol L^–1^ aqueous KOH solution shows remarkable performance (1000 mA cm^–2^ at 1.85 V) and durability exceeding 12000 h. This seems to support that a hybrid solution that combines materials, structures, morphologies, and support electrolyte systems from the different approaches is a possible way forward in the development of separators for the next generation alkaline water electrolysis technology. Separators made of highly alkali-resistant materials with a high volume fraction of narrow electrolyte-filled channels could potentially combine high conductivity with excellent H_2_ barrier characteristics and good stability, and could therefore be a rational way forward.

**Table 2:** Key Characteristics of the Three Separator Technologies

	**Porous diaphragm**	**AEMs**	**Ion-solvating membrane**
**Polymer type**	Any hydrophilic polymer or hydrophilic composite	Cationic polymers	Any anionic, cationic, or neutral hydrophilic polymer
**Pure water operation**	Not possible	Possible	Not possible
**Ionomer in catalyst layer**	No need	Mandatory for pure water operation	No need
**H**_**2**_**crossover**	Significant	Expected lower	Lower
**Unbalanced pressure**	Not possible	Possible	Possible
**High pH electrolyte**	Yes	No need	Yes
**Durability**	High	To be proven	To be proven
**State-of-art**	Mature	Immature	Immature
